# Supplementary material for: The Systems Biology Research Tool: evolvable open-source software
Source: BMC Syst Biol. 2008 Jun 29;2:55. doi: 10.1186/1752-0509-2-55 (PMC2446383; doi:10.1186/1752-0509-2-55)
Supplement: Additional file 1 — SBRT Archive. An archive of the current version of the Systems Biology Research Tool. [file 1752-0509-2-55-S1.zip › sbrt-1.4.0/doc/developers_guide/api/sbrt/shell/ApplicationException.html]

ApplicationException


|  |  |  |
| --- | --- | --- |
| **Overview** \| **Package** \| **Class** \| **Use** \| **Tree** \| **Deprecated** \| **Index** \| **Help** | |  |
| PREV CLASS   **NEXT CLASS** | **FRAMES**    **NO FRAMES**     **All Classes** |  |
| SUMMARY: NESTED \| FIELD \| CONSTR \| METHOD | DETAIL: FIELD \| CONSTR \| METHOD |  |


---


## sbrt.shell Class ApplicationException

```
java.lang.Object
  java.lang.Throwable
      java.lang.Exception
          java.lang.RuntimeException
              sbrt.shell.ApplicationException
```

**All Implemented Interfaces:**: java.io.Serializable

**Direct Known Subclasses:**: FormatException

---

``` public class ApplicationException extends java.lang.RuntimeException ```

This class is the superclass of those exceptions that are
expected to be thrown from the SBRT shell. If an exception
is thrown from the shell that is not a subclass of this
class, it is indicative of a bug.

**Author:**
:   This class was written and documented by
    Jeremiah Wright while in the Wagner lab.

**See Also:**: Serialized Form

---

**Constructor Summary**

`ApplicationException(java.lang.String message)`
  
          Constructs a new process exception with
the provided error message.

`ApplicationException(java.lang.String message,
java.lang.Throwable cause)`
  
          Constructs a new process exception with
the provided error message and cause.


| **Method Summary** | |
| --- | --- |
| `java.lang.String` | `getMessage()`             Returns the error message of this process exception. |

**Methods inherited from class java.lang.Throwable**

`fillInStackTrace, getCause, getLocalizedMessage, getStackTrace, initCause, printStackTrace, printStackTrace, printStackTrace, setStackTrace, toString`

**Methods inherited from class java.lang.Object**

`clone, equals, finalize, getClass, hashCode, notify, notifyAll, wait, wait, wait`

**Constructor Detail**

### ApplicationException

```
public ApplicationException(java.lang.String message)
```

:   Constructs a new process exception with
    the provided error message.

    **Parameters:**: `message` - a complete, informative, well-formed sentence describing the error that ocurred (which is saved for later retrieval by the `Throwable.getMessage()` method).

---


### ApplicationException

```
public ApplicationException(java.lang.String message,
                            java.lang.Throwable cause)
```

:   Constructs a new process exception with
    the provided error message and cause.

    **Parameters:**: `message` - a complete, informative, well-formed sentence describing the error that ocurred (which is saved for later retrieval by the `Throwable.getMessage()` method).: `cause` - the cause (which is saved for later retrieval by the `Throwable.getCause()` method). (A null value is permitted, and indicates that the cause is nonexistent or unknown.)


**Method Detail**

### getMessage

```
public java.lang.String getMessage()
```

:   Returns the error message of this process
    exception.

    :   **Overrides:**: `getMessage` in class `java.lang.Throwable`
    :   **Returns:**: the error message of this process exception.


---


|  |  |  |
| --- | --- | --- |
| **Overview** \| **Package** \| **Class** \| **Use** \| **Tree** \| **Deprecated** \| **Index** \| **Help** | |  |
| PREV CLASS   **NEXT CLASS** | **FRAMES**    **NO FRAMES**     **All Classes** |  |
| SUMMARY: NESTED \| FIELD \| CONSTR \| METHOD | DETAIL: FIELD \| CONSTR \| METHOD |  |


---
